# Developing the modified 4-item version of perceived stress scale for functional dyspepsia

**DOI:** 10.1186/s12876-023-02728-0

**Published:** 2023-03-29

**Authors:** Limei Du, Guizhen Yong, Ping Wang, Xi Wang, Wen Ming, Guobin He

**Affiliations:** 1 Department of Gastroenterology, Affiliated Hospital of North Sichuan Medical College, No. 67 Wenhua Road, Shunqing, Nanchong, 637000 Sichuan China; 2 Department of Nursing, Affiliated Hospital of North Sichuan Medical College, No. 67 Wenhua Road, Shunqing, Nanchong, 637000 Sichuan China

**Keywords:** Functional dyspepsia, Perceived stress scale, Modified 4-item version, Psychological stress, Development, Somatization, Quality of life

## Abstract

**Background:**

To develop the modified 4-item version of Perceived Stress Scale (PSS) with a better reliability and validity than the 4-item version of PSS (PSS-4) in evaluating psychological stress in patients with functional dyspepsia (FD). The present study also aimed to explore the correlation between dyspepsia symptoms severity (DSS), anxiety, depression, somatization, quality of life (QoL), and psychological stress assessed by two approaches in FD.

**Methods:**

A total of 389 FD patients who met the Roman IV criteria completed the 10-item version of the PSS (PSS-10), and 4/10 items were selected by five methods, such as Cronbach’s coefficient, exploratory factor analysis (EFA), correlation coefficient, discrete degree, and item analysis, to develop the modified PSS-4. The reliability and validity of the modified PSS-4 and the PSS-4 were compared by internal consistency, EFA, and confirmatory factor analysis (CFA). The correlation between psychological stress assessed by two approaches and DSS, anxiety, depression, somatization, and QoL was explored by Pearson’s correlation coefficient and multiple linear regression analysis.

**Results:**

Cronbach’s α coefficient of the modified PSS-4 and the PSS-4 was 0.855 and 0.848, respectively, and a common factor was extracted. The cumulative contribution rate of one factor to the overall variance for the modified PSS-4 and the PSS-4 was 70.194% and 68.698%, respectively. The model used for the modified PSS-4 showed that the values of the goodness-of-fit index (GFI) and the adjusted GFI (AGFI) were 0.987 and 0.933, respectively, indicating that the model fitted well. Psychological stress was correlated to DSS, anxiety, depression, somatization, and QoL as assessed by the modified PSS-4 and PSS-4. Multiple linear regression analysis revealed that psychological stress was correlated to somatization, as assessed by the modified PSS-4 (*β* = 0.251, *P* < 0.001) and PSS-4 (*β* = 0.247, *P* < 0.001). Psychological stress, DSS, and somatization were correlated to QoL, as assessed by the modified PSS-4 (*β* = 0.173, *P* < 0.001) and the PSS-4 (*β* = 0.167, *P* < 0.001).

**Conclusions:**

The modified PSS-4 showed better reliability and validity, and psychological stress had a greater effect on the somatization and QoL of FD patients assessed by the modified PSS-4 than PSS-4. These findings were helpful for further investigation of the clinical application of the modified PSS-4 in FD.

**Supplementary Information:**

The online version contains supplementary material available at 10.1186/s12876-023-02728-0.

## Background

### Definition and subtypes of functional dyspepsia (FD)

FD is a chronic gastrointestinal (GI) disorder defined by upper abdominal symptoms that originate from the gastroduodenal region with no structural disease detected in routine investigation, including upper GI endoscopy; it is characterized by chronicity, persistence, and easy recurrence [1, 2]. Rome IV criteria divides FD into three subtypes: postprandial distress syndrome (PDS) with postprandial fullness or early satiation, epigastric pain syndrome (EPS) with epigastric pain or epigastric burning, and a subtype with overlapping PDS and EPS features [3]. As one of the most prevalent functional gastrointestinal disorders (FGIDs) in the digestive system, FD has a high prevalence. The global prevalence of uninvestigated dyspepsia is estimated to be 21%, and the prevalence of FD is approximately 16%, but it might vary across countries and according to the criteria used to define its presence [4, 5]. Data from the USA, Canada, and UK showed a 10% prevalence of FD in the adult population using the Rome IV criteria, with a similar distribution pattern across different regions (61% PDS, 18% EPS, and 21% overlap) [6], while the prevalence was 12.7% (39% PDS, 33% EPS, and 28% overlap) in Bulgarian adults [7].

### Pathogenesis and stress of functional dyspepsia

Symptoms of FD can be caused by disturbed gastric motility (for example, inadequate fundic accommodation or delayed gastric emptying), gastric sensation (for example, sensations associated with hypersensitivity to gas and abdominal distension), gastroduodenal mucosal inflammation, local or systemic immune activation, *Helicobacter pylori* (*H.* pylori) infection, altered gut microbiota, and psychological factors [3, 8]. For the occurrence and development of FD, the influence of mental disorders (such as anxiety and depression) has been under intensive focus, but less attention has been paid to psychological stress. Some studies showed that psychological stress plays a critical role in FD [9, 10]. Therefore, evaluating the psychological stress of FD patients has vital implications for the treatment and prognosis of the disease.

### Development and limitations of perceived stress scale (PSS)

PSS, is one of the most widely used stress perception assessment instruments worldwide [11]. The scale was originally developed in 1983 by Cohen et al. [12] to assess the degree of stress that was experienced in unpredictable, out-of-control, and overloaded situations. PSS is a self-reported scale with three versions: 14-item scale (PSS-14), 10-item scale (PSS-10), and 4-item scale (PSS-4). PSS-10 has better reliability and validity than the other two versions [13]. As a tool for subjective measurement of stress, PSS has been translated into multiple languages, such as Japanese, Greek, French, Korean, and Chinese, and is widely used to study psychological stress in various populations [14–20]. However, few studies have used PSS to explore the correlation between psychological stress and FD. In clinical research, when data need to be collected over the phone or to quickly evaluate psychological stress in patients within a limited period, PSS-14 and PSS-10 have many items and are time-consuming, which might lead to patients not answering seriously or even refusing to continue to answer, thus reducing the authenticity of the answers. Therefore, the PSS-4 was developed for easy usage in case of time constraints on data collection (such as during telephone interviews) [12]. It has four items and is less time-consuming, making it suitable for rapid clinical data collection; however, the scale has lower reliability and validity than the other two versions and has not been studied in FD. Therefore, the present study aimed to simplify and revise the PSS-10 items by applying five-item screening methods to develop the modified PSS-4, compare the reliability and validity with the PSS-4, and explore the correlation between psychological stress assessed by two approaches and based on the symptoms of FD. Thus, we hope that the current findings will contribute to further research on the clinical application of the modified PSS-4 in FD patients.

## Patients and methods

### Patients

A total of 403 patients initially diagnosed with FD in the outpatient clinic of gastroenterology at the Affiliated Hospital of North Sichuan Medical College from September 2020 to October 2021 were selected in the current study; the minimum sample size was 300 patients to achieve a 95% confidence interval (CI) to equally split the chance according to a previously mentioned formula [21]. Inclusion criteria: (1) patients who have one or more dyspepsia symptoms (including postprandial fullness, early satiation, epigastric pain, or epigastric burning) according to the Roman IV criteria [3] for the past 3 months with onset ≥ 6 months before diagnosis; (2) age was 18–70 years; (3) patients with a certain cognitive ability and can complete the questionnaire independently. Exclusion criteria: (1) upper GI organic diseases, including esophagitis, peptic ulcer, and Barrett’s esophagus, which were diagnosed by gastroscopy or upper GI examination; (2) patients with systemic diseases, metabolic diseases, or malignant tumors diagnosed by auxiliary examinations, including blood routine, biochemical examinations, thyroid function, glycosylated hemoglobin, and B-scan ultrasound; (3) patients with irritable bowel syndrome; (4) patients with gastroesophageal reflux disease with acid reflux and heartburn as primary symptoms; (5) patients with a history of abdominal surgery; (6) patients who had currently ingested anticholinergic drugs, antispasmodic pain relievers, nonsteroidal anti-inflammatory drugs, and hormones; (7) pregnant and lactating female patients. Finally, 389 FD patients were included in the study after they signed the written informed consent form.

## Instruments

### PSS

The PSS-10 (Appendix) was used to scientifically evaluate the individual’s perception of stress in the last month [13]. The scale consisted of ten items, and each item was scored on a 5-point Likert scale ranging from 0 (“never”) to 4 (“very often”). The scores ranged from 0 to 40, with higher scores indicating more perceived stress. The PSS-4, the briefest version of the PSS, included only four items (Appendix), and provided a valuable tool for data collection over the phone [12]. The scale made it feasible to repeat measurements of perceived stress in large samples; the scores range from 0 to 16, with higher scores indicating more perceived stress.

### Generalized anxiety disorder-7 (GAD-7)

The GAD-7 has good reliability as well as criterion, construct, factorial, and procedural validity. It consists of seven items for detecting the frequency of anxiety symptoms during the lately two weeks [22]. The response options were “not at all,” “several days,” “more than half the days,” and “nearly every day,” scored as 0, 1, 2, and 3, respectively. Scores range from 0 to 21, and high scores indicate severe anxiety symptoms. Patients with a score ≥ 10 were diagnosed to be in a status of anxiety [23]. The GAD-7 has demonstrated good reliability and validity in the Chinese population [24].

### Health questionnaire depression nine-Item Scale (PHQ-9)

The PHQ-9 is a self-reported version of the Primary Care Evaluation of Mental Disorders (PRIME-MD). It consists of nine items for measuring the frequency of depressive symptoms within two weeks [25]. Each item ranges from 0 (“not at all”) to 3 (“nearly every day”). The total score represents the severity of depression symptoms ranging from 0 to 27, and patients with a score ≥ 10 were diagnosed to have depression [22]. The Chinese PHQ-9 has demonstrated good reliability and validity in the general population [26].

### Health questionnaire depression fifteen-item scale (PHQ-15)

The PHQ-15 was a somatic symptom subscale derived from the full PHQ and a brief, self-administered questionnaire that may be useful in screening or somatization and in monitoring somatic symptom severity in clinical practice and research. It consists of 15 items that assess the extent to which a patient has been affected by each item over the past 4 weeks [27]. Each symptom was scored from 0 (“not bothered at all”) to 2 (“bothered a lot”), and the scores ranged from 0 to 30, with higher scores indicating severe somatic symptoms.

### Dyspepsia symptoms score (DSS)

DSS was designed to quantify the severity of dyspepsia symptoms in FD patients with epigastric pain, epigastric burning, postprandial fullness, early satiation, bloating, nausea, vomiting, and belching over the past two weeks [28]. Each symptom was scored from 0 (“not at all”) to 3 (“significantly affect life and work”). The total score ranges from 0 to 24, with higher scores indicating severe symptoms.

### 10-item short form of the nepean dyspepsia index (SF-NDI)

SF-NDI was originally developed for quality of life (QoL) assessment in patients with FD over the past two weeks. It includes five subscales, such as tension, interference with daily activities, eating/drinking, knowledge/control, and work/study, and each subscale contains two items. The items were measured by a 5-point graded Likert scale from 1 to 5. A total sum score for QoL and a sum score for each of the five subscales were calculated by adding up scores of each item (range of each subscale, 2–10) [29, 30]; higher scores indicate poor functioning or symptoms.

### Statistical analysis

We used SPSS 23.0 software and AMOS 24.0 software for statistical analysis. Continuous variables were expressed as mean ± standard deviation (SD), t-test was used for comparison between two groups, and one-way analysis of variance (ANOVA) was used for comparison of three groups or more. The categorical variables were expressed as rates (%). The reliability of the two approaches was analyzed by coefficient α, and the validity was analyzed by exploratory factor analysis (EFA) and confirmatory factor analysis (CFA). Sample characteristics were analyzed by descriptive analysis. Pearson’s correlation coefficient was used for correlation analysis. Multiple linear regression analysis was used to analyze the factors influencing somatization and QoL in FD patients. All statistical tests were two-tailed, and the results were significant at *P* < 0.05.

## Results

### Demographic characteristics of the sample

The current sample consisted of 37.8% (147/389) males and 62.2% (242/389) females. The mean age of the patients was 46.3 ± 10.1 (range 18–69)-years-old. The educational levels were divided into five groups: 4.9% (19/389) below primary school, 22.6% (88/389) primary school, 39.6% (154/389) middle school, 15.4% (60/389) high school, 17.5% (68/389) college or above. FD was divided into three subtapes: 34.2% (133/389) EPS, 16.5% (64/389) PDS, 49.3% (192/389) overlap. The study showed that female patients were more prone to FD than males, and the education level of patients was low, mainly middle school. Moreover, the overlap was common. Table 1 presents the demographic characteristics of the sample.

**Table 1:** Demographic analysis of the modified PSS-4 and PSS-4

	PSS-4				Modified PSS-4			
Information	Mean	SD	t/F	*P*	Mean	SD	t/F	*P*
Gender			-0.143	0.886			-0.058	0.954
Male (n = 147)	6.79	3.29			6.48	3.41		
Female (n = 242)	6.83	2.89			6.50	3.17		
Educational levels			0.744	0.562			0.399	0.810
Below primary school (n = 19)	7.74	3.21			7.21	3.21		
Primary school (n = 88)	6.85	2.78			6.47	3.01		
Middle school (n = 154)	6.84	3.06			6.53	3.28		
High school (n = 60)	6.87	3.03			6.55	3.31		
College or above (n = 68)	6.41	3.30			6.18	3.48		
FD subtypes			6.270	0.002			5.365	0.005
EPS (n = 133)	6.56	3.23			6.18	3.41		
PDS (n = 64)	5.88	2.87			5.61	3.39		
Overlap (n = 192)	7.31	2.88			6.99	3.02		

FD, functional dyspepsia; EPS, epigastric pain syndrome; PDS, postprandial discomfort syndrome; Overlap, subtype with overlapping PDS and EPS features; SD, standard deviation

### Items were selected by five methods for developing the modified PSS-4

Cronbach’s α coefficient: The internal consistency of the scale (i.e. the reliability of the scale) was evaluated by Cronbach’s α. The higher the α, the better the reliability and the internal consistency of the scale. α > 0.7 indicates good reliability of the scale [31, 32]. Also, the α of the initial total scale was calculated; an increase in the value after deleting an item indicated that the existence of the item reduces the internal consistency, and deleting the specific item improves the reliability of the scale [33, 34]. In the present study, Cronbach’s α of the PSS-10 was 0.897. After deleting items 7 and 9, α was higher than the initial value.

Exploratory factor analysis: The Kaiser–Meyer–Olkin (KMO) and Bartlett’s sphericity test evaluated whether the data were suitable for factor analysis. The value of KMO > 0.7 indicated a satisfactory effect of factor analysis. In Bartlett’s sphericity test, *P* < 0.05 indicated a correlation between items, which was suitable for factor analysis. Then, principal component and varimax rotation were utilized to examine the dimensionality of the items, and an item with a loading factor value < 0.4 was excluded [35, 36]. In this study, the values of the PSS-10 were 0.926 and 1872.622 (*P* < 0.001) in KMO and Bartlett’s test, indicating suitability for factor analysis, which showed that the factor loading of each item was > 0.4.

Correlation coefficient method: The higher the correlation between the score of each item and the total score of the scale, the greater the consistency between the characteristics measured by each item and the total score of the scale. Consequently, an item with a correlation coefficient > 0.4 was selected [37]. In the present study, the correlation coefficient of each item was > 0.4.

Discrete degree method: SD was used to measure the degree of dispersion. The lower degree of dispersion of the selected item, the worse the evaluation [38]. Herein, an item with SD < 0.5 was excluded.

Item analysis method: Descriptive analysis was performed to analyze the concentration of answers to the item. An option in a certain item frequency value was selected > 80% or the sum of the selected frequency of any two options was < 10%, indicating that the answers had a significantly skewed distribution and the item should be considered for exclusion [39]. In the current study, the sum of selection frequency of the two options in items 1, 2, 3, 5, 7, and 9 was 5.4%, 7.7%, 6.1%, 9.5%, 3.9%, and 5.7%, respectively, all of which were < 10% (Table 2).

**Table 2:** Results of item analysis method

Items	Selected frequency (%)				
	0-point	1-point	2-point	3-point	4-point
1	4.4	26.7	45.8	22.1	1.0
2	5.9	23.9	43.2	25.2	1.8
3	3.1	22.4	43.7	27.8	3.0
4	14.9	44.2	27.2	12.6	1.1
5	14.4	28.0	48.1	9.0	0.5
6	19.0	26.5	37.5	13.1	3.9
7	24.9	42.9	28.3	3.6	0.3
8	17.2	21.9	47.6	13.2	0.1
9	3.3	16.7	47.3	30.3	2.4
10	8.2	22.9	38.0	27.5	3.4

### Modified PSS-4

The PSS-10 was screened by the above five methods, and the screening plan was summarized in Table 3. Finally, items 4, 6, 8, and 10 were retained to develop the modified PSS-4 (see Appendix).

**Table 3:** Item screening methods of the modified PSS-4

Items	Cronbach’s α coefficient	Factor analysis	Correlation coefficient	Discrete degree	Item analysis
1	0.887	0.707	0.705	0.833	(×)
2	0.883	0.776	0.768	0.893	(×)
3	0.885	0.739	0.737	0.864	(×)
4	0.884	0.754	0.748	0.925	(√)
5	0.885	0.738	0.730	0.866	(×)
6	0.887	0.723	0.733	1.060	(√)
7	0.901(×)	0.487	0.512	0.830	(×)
8	0.878	0.830	0.822	0.927	(√)
9	0.898(×)	0.529	0.545	0.828	(×)
10	0.873	0.877	0.871	0.983	(√)

(×) means to remove the item; (√) means to retain the item

### Reliability analysis of the modified PSS-4 and PSS-4

We used Cronbach’s α coefficient and split-half reliability to analyze the reliability of the two scales: The Cronbach’s α of the modified PSS-4 and the PSS-4 was 0.855 and 0.848, respectively. The average correlation coefficient among the items was 0.600 and 0.581, respectively. The range of the correlation coefficient between each item and the total scale score was 0.799–0.904 and 0.781–0.886, respectively. Moreover, after removing any item in the modified PSS-4, Cronbach’s α of the scale was 0.835, 0.849, 0.811, and 0.764, respectively, which was still < 0.855. Furthermore, the two scales were randomly divided into parts by SPSS 23.0 software. The Guttman coefficient > 0.7 indicated a good internal consistency of the scale. The results showed that the Guttman coefficient of the modified PSS-4 and PSS-4 was 0.865 and 0.855, respectively, indicating that the reliability of the modified PSS-4 was better than that of PSS-4.

## Validity analysis of the modified PSS-4 and PSS-4

### Criterion validity

The correlation coefficient between the total score of the modified PSS-4 and PSS-4 and PSS-10 was (r = 0.948, *P* < 0.001) and (r = 0.942, *P* < 0.001). The correlation coefficient between the total score of the modified PSS-4 and PSS-4 was (r = 0.931, *P* < 0.001).

### Structural validity

EFA revealed that the values of the modified PSS-4 were 0.790 and 736.810 (*P* = 0.000) in KMO and Bartlett’s test, while those of the PSS-4 were 0.778 and 679.027 (*P* = 0.000), respectively. The cumulative contribution rate of the modified PSS-4 was 70.194%, and the factor loading of each item was > 0.4. In addition, the cumulative contribution rate of the PSS-4 was 68.698%. For the CFA, chi-square value (*x*)^*2*^, GFI, AGFI, normative fit index (NFI), comparative fit index (CFI), root mean square residual (RMR), and root mean square error of approximation (RMSEA) were used to evaluate the model fit obtained by CFA. The smaller the value of *x*^2^, the better the model fit. GFI, AGFI, NFI, and CFI > 0.9 indicated that the model was well. Moreover, RMR and RMSEA < 0.08 indicated that the model was acceptable, and < 0.05 indicated that the model was well-established [40–42]. The results of CFA are shown in Table 4.

**Table 4:** Confirmatory factor analysis of the modified PSS-4 and the PSS-4

Scale	*x* ^*2*^	*x*^*2*^/df	GFI	AGFI	NFI	CFI	RMR	RMSEA
Modified PSS-4	10.197	5.099	0.987	0.933	0.986	0.989	0.020	0.103
PSS-4	20.577	10.289	0.974	0.871	0.970	0.973	0.028	0.155

CFA, confirmatory factor analysis; *x*^*2*^, chi-square value; df, degrees of freedom; *x*^2^/df, chi-square degree of freedom ratio; GFI, goodness-of-fit index; AGFI, adjusted goodness-of-fit index, NFI, normative fit index; CFI, comparative fit index; RMR, root mean square residual; RMSEA, root mean square error of approximation

### Demographic analysis of the modified PSS-4 and PSS-4

The average score of the modified PSS-4 and PSS-4 was 6.49 ± 3.26 and 6.82 ± 3.04, respectively, indicating a significant difference in FD subtypes (*P* < 0.05). The post-hoc test demonstrated a significant difference between PDS and overlap group (*P* = 0.001), EPS and overlap group (*P* = 0.026), but no significant difference between PDS and EPS (*P* = 0.136) in the scores assessed by PSS-4. Among the scores assessed by the modified PSS-4, a significant difference was detected between PDS and overlap group (*P* = 0.003), EPS and overlap group (*P* = 0.026), but no significant difference was observed between PDS and EPS (*P* = 0.245). However, no significant differences were detected in gender and education levels (*P* > 0.05). The demographic analysis is shown in Table 1.

### Correlation analysis between psychological stress and symptoms of FD

The correlation coefficient between psychological stress assessed by the modified PSS-4 and somatization and QoL (r = 0.302, *P* < 0.001) and (r = 0.255, *P* < 0.001) was higher than that of PSS-4 (Table 5). Further correlation analysis unveiled that the correlations between somatization and QoL were associated with psychological stress assessed by the two approaches in patients with EPS, PDS, and Overlap. The results are shown in Tables 6 and 7.

**Table 5:** Correlation analysis between stress and dyspepsia symptoms, anxiety, depression, somatization, and QoL

Variables	PSS-4		Modified PSS-4	
Anxiety	r = 0.573	*P* < 0.001	r = 0.552	*P* < 0.001
Depression	r = 0.413	*P* < 0.001	r = 0.390	*P* < 0.001
DSS	r = 0.158	*P* < 0.001	r = 0.148	*P* = 0.003
FD subtypes	r = 0.177	*P* < 0.001	r = 0.164	*P* = 0.001
Somatization	r = 0.301	*P* < 0.001	r = 0.302	*P* < 0.001
QoL	r = 0.250	*P* < 0.001	r = 0.255	*P* < 0.001

DSS, dyspepsia symptoms score; FD, functional dyspepsia; QoL, quality of life

**Table 6:** Correlation analysis between psychological stress and somatization

Scale	FD (n = 389)	EPS (n = 133)	PDS (n = 64)	Overlap (n = 192)
Modified PSS-4				
r	0.302	0.273	0.098	0.464
*P*	< 0.001	0.001	0.439	< 0.001
PSS-4				
r	0.301	0.262	0.072	0.482
*P*	< 0.001	0.002	0.571	< 0.001

FD, functional dyspepsia; EPS, epigastric pain syndrome; PDS, postprandial discomfort syndrome

**Table 7:** Correlation analysis between psychological stress and quality of life

Scale	FD (n = 389)	EPS (n = 133)	PDS (n = 64)	Overlap (n = 192)
Modified PSS-4				
r	0.225	0.197	0.190	0.413
*P*	< 0.001	0.023	0.132	< 0.001
PSS-4				
r	0.220	0.212	0.176	0.405
*P*	< 0.001	0.014	0.163	< 0.001

FD, functional dyspepsia; EPS, epigastric pain syndrome; PDS, postprandial discomfort syndrome

### Linear regression analysis of the effect of psychological stress on somatization

In this study, somatization was used as a dependent variable, whereas psychological stress, anxiety, depression, and DSS were used as independent variables, and the entry method was used for linear regression analysis. The study revealed that psychological stress was not detected as a factor influencing somatization. However, after removing anxiety and depression, the study identified psychological stress and DSS as the influencing factors (Table 8). The adjusted R^2^ for the modified PSS-4 and PSS-4 regression equation was 0.203 and 0.201, respectively.

**Table 8:** Linear regression analysis of the influence on the somatization after removing anxiety and depression

Variable	Unstandardized coefficients	Standardized coefficients	t	*P* value	Adjusted R^2^
Model 1					0.203
Modified PSS-4	0.250	0.251	5.471	< 0.001	
DSS	0.432	0.345	7.528	< 0.001	
Model 2					0.201
PSS-4	0.264	0.247	5.373	< 0.001	
DSS	0.429	0.343	7.462	< 0.001	

R^2^, the fit degree of the model; DSS, dyspepsia symptoms score

### Linear regression analysis of the effect of psychological stress on QoL

In this study, QoL was used as a dependent variable, while psychological stress, anxiety, depression, DSS, FD subtypes, and somatization were independent variables. This study deduced that psychological stress was not a factor influencing the QoL. However, after removing anxiety and depression, psychological stress, DSS, FD subtypes, and somatization were still identified as influencing factors (Table 9). The adjusted R^2^ for the modified PSS-4 and PSS-4 was 0.261 and 0.259, respectively.

**Table 9:** Linear regression analysis of the influence on the QoL after removing anxiety and depression

Variable	Unstandardized coefficients	Standardized coefficients	t	*P* value	Adjusted R^2^
Model 1					0.261
Modified PSS-4	0.237	0.173	3.701	< 0.001	
DSS	0.487	0.283	5.978	< 0.001	
Somatization	0.297	0.216	4.388	< 0.001	
FD subtypes	-0.924	− 0.154	-3.435	0.001	
Model 2					0.259
PSS-4	0.245	0.167	3.558	< 0.001	
DSS	0.484	0.281	5.924	< 0.001	
Somatization	0.300	0.219	4.438	< 0.001	
FD subtypes	-0.932	− 0.155	-3.448	0.001	

R^2^, the fit degree of the model; DSS dyspepsia symptom score

## Discussion

Although a common FGIDs in the digestive system, the pathogenesis of FD has not yet been clarified. FD is difficult to cure due to its diverse clinical manifestations and characteristics, such as chronic, persistent, and easy recurrence features, which cause a severe physical and psychological burden to patients [43]. The emerging bio-social-psychological medical model has confirmed the occurrence and development of many diseases are related to biological, social, and psychological factors.

Several studies have shown that psychological stress is strongly associated with the symptoms of FD [9, 44, 45]. For example, stress is an independent risk factor for FD and the incidence rates of FD rise with increasing stress levels [10]. Furthermore, the overlap of the central and peripheral stress systems with key pathways of the brain-gut axis further support the correlation between stress and FD [9]. Therefore, assessing psychological stress in patients with FD may have important implications for the treatment and prognosis of the disease.

PSS-4 is a short form for subjective measurement of stress, which is suitable for collecting data by phone or quickly assessing psychological stress of elderly patients in outpatient clinics. However, it is not a specific scale for assessing psychological stress in FD, and many studies have shown that the overall Cronbach’s α for PSS-4 is low, ranging from 0.55 to 0.68; and it has not been studied in the FD population [16, 46]. Therefore, developing a short scale with high reliability and validity to rapidly assess the psychological stress of FD patients is essential.

In this study, the items of PSS-10 were screened by five-item screening methods to develop the modified PSS-4; the scientific rationality of the modified version was confirmed. From the perspective of reliability analysis: Firstly, Cronbach’s α of the modified PSS-4 and PSS-4 was 0.855 and 0.848, respectively. Although worldwide studies have shown that Cronbach’s α of the PSS-4 ranged from 0.55 to 0.68, these studies have not been conducted in the population of FD, and different diseases may have varied outcomes. Secondly, the average and the range of correlation coefficient of each item and the total score of the modified PSS-4 were higher than PSS-4. These results indicated that the internal consistency of the modified PSS-4 may be better than that of PSS-4. From the perspective of validity analysis: Firstly, the PSS-10 has been used as a clinical tool for measuring psychological stress in various populations, and its reliability and validity have been verified. The high correlation coefficient between the total score of the modified PSS-4 and PSS-10 indicated that the modified PSS-4 was highly correlated with PSS-10. Secondly, EFA showed that the factors of the modified PSS-4 had a higher cumulative contribution rate to the overall variance. Thirdly, CFA showed that the one-factor model fit of the modified PSS-4 may be better than that of PSS-4. These results suggested that the validity of the modified PSS-4 may be better than the PSS-4. Together, the reliability and validity of the modified PSS-4 may be better than that of PSS-4.

The analysis of the application of the modified PSS-4 in FD patients revealed the following: Firstly, the demographic analysis found that psychological stress, assessed by two approaches, was related to somatization and QoL in EPS and Overlap but not PDS, which could be because the majority of patients presented EPS and Overlap. Secondly, the study identified a higher correlation between the modified PSS-4 and somatization and QoL compared to PSS-4, indicating that the modified PSS-4 can evaluate the correlation between psychological stress and somatization and QoL. Thirdly, linear regression analysis affecting somatization and QoL showed that psychological stress was not an influencing factor, indicating that anxiety and depression had a greater impact on FD patients than psychological stress. However, the influence of psychological stress is common in the clinic. Further analysis after removing anxiety and depression showed that psychological stress is a major influencing factor, prompting additional investigation. Fourthly, since somatization is a risk factor for impaired QoL in FD patients [47], it was included in the regression analysis as an independent variable in this study. Finally, linear regression analysis showed that psychological stress, assessed by two approaches, affected somatization and QoL, and the modified PSS-4 had a greater impact on FD than PSS-4.

The strengths of our study were as follows: the reliability and validity of the modified PSS-4 may be better than that of PSS-4 in FD patients. In order to collect data by quickly assessing the psychological stress of patients in outpatient clinics, the modified PSS-4 is a better tool than PSS-4. Nevertheless, the study has some limitations. Firstly, the patients in this study may have visited primary hospitals many times due to severe symptoms and significant psychological burden, resulting in selection bias of the subjects. Secondly, although the fitting of the modified PSS-4 was better than that of PSS-4, the confirmatory factor analysis study showed that the structural validity of the two scales did not fulfill the criteria for scale validity. Thirdly, we did not perform a retesting reliability analysis on the modified PSS-4 in FD patients. Fourthly, the study did not explore the optimal score cutoff point of the modified PSS-4 for assessing psychological stress in patients with FD. Thus, the reliability of the scale needs to be substantiated further. Finally, the clinical application of the modified PSS-4 needs be verified by further studies.

## Conclusions

The current study suggested that the reliability and validity of the modified PSS-4 may be better than PSS-4. Furthermore, psychological stress assessed by the modified PSS-4 may correlate better with somatization and QoL and exert a marked effect on FD patients than PSS-4. These findings are helpful for further research on the clinical application of the modified PSS-4 in FD.

## Electronic supplementary material

Below is the link to the electronic supplementary material.

**Appendix: Items and Instructions for Perceived Stress Scale**

## Data Availability

The data supporting the current findings are available from the corresponding author upon reasonable request.

## Abbreviations

FD: functional dyspepsia
The modified PSS-4: The modified 4-item version of the Perceived Stress Scale
PSS-4: 4-item version of the Perceived Stress Scale
PSS-10: The 10-item version of the Perceived Stress Scale
EPS: Epigastric pain syndrome
PDS: Postprandial distress syndrome
Overlap: A subtype with overlapping PDS and EPS features
QoL: Quality of life
CFA: Confirmatory factor analysis
*x*^*2*^: Chi-square value
df: Degrees of freedom
*x*^2^/df: The chi-square degree of freedom ratio
GFI: Goodness-of-fit index
AGFI: Adjusted goodness-of-fit index
NFI: Normative fit index
CFI: Comparative fit index
RMR: Root mean square residual
RMSEA: Root mean square error of approximation
PHQ-15: Health Questionnaire Depression Fifteen-Item scale
GAD-7: Generalized Anxiety Disorder-7
PHQ-9: Health Questionnaire Depression Nine-Item scale
SF-NDI: The 10-item Short Form of the Nepean Dyspepsia Index
DSS: Dyspepsia symptom severity
SD: Standard deviation
