# Supplementary material for: Developing the modified 4-item version of perceived stress scale for functional dyspepsia
Source: BMC Gastroenterol. 2023 Mar 29;23:97. doi: 10.1186/s12876-023-02728-0 (PMC10061803; doi:10.1186/s12876-023-02728-0)
Supplement: Supplementary file 1 — Appendix: Items and Instructions for Perceived Stress Scale [file 12876_2023_2728_MOESM1_ESM.docx]

**Appendix**

**Items and Instructions for Perceived Stress Scale**

The questions in this scale ask you about your feelings and thoughts during the last month. In each case, you will be asked to indicate how often you felt or thought a certain way. For each question choose from the following alternatives:

0. never

1. almost never

2. sometimes

3. fairly often

4. very often

PSS-10

1 Upset by something happening unexpectedly?

2 Unable to control the important things in your life?

3 Nervous and stressed?

4 Confident about your ability to handle your personal problems?

5 Things were going your way?

6 Could not cope with all the things that you had to do?

7 Dealt successfully with irritating life hassles?

8 You were on top of things?

9 Angered because of things that happened that were outside your control?

10 Difficulties were pilling up so high that you could not overcome them?

PSS-4

2 Unable to control the important things in your life?

4 Confident about your ability to handle your personal problems?

5 Things were going your way?

10 Difficulties were piling up so high that you could not overcome them?

The modified PSS-4

4 Confident about your ability to handle your personal problems?

6 Could not cope with all the things that you had to do?

8 You were on top of things?

10 Difficulties were pilling up so high that you could not overcome them?
